# Intraplacental choriocarcinoma coexisting with fetomaternal hemorrhage: Case report, chemotherapy management, and literature review

**DOI:** 10.1097/MD.0000000000009977

**Published:** 2018-04-06

**Authors:** Qin She, Zhi Cheng, Darine El-Chaar, Feng Luo, Xiaoyan Guo, Shi Wu Wen

**Affiliations:** a Department of Obstetrics and Gynecology, the Six Affiliated Hospital, Guangzhou Medical University, Qingyuan, Guangdong Province, China; b OMNI Research Group, Department of Obstetrics and Gynecology, University of Ottawa; c Ottawa Hospital Research Institute Clinical Epidemiology Program; d Department of Obstetrics, Gynecology and Newborn Care, The Ottawa Hospital, Ottawa, ON, Canada; e Department of Pathology, the Six Affiliated Hospital, Guangzhou Medical University, Qingyuan, Guangdong Province, China; f School of Epidemiology, Public Health, and Preventive Medicine, University of Ottawa Faculty of Medicine, Ottawa, ON, Canada.

**Keywords:** beta-human chorionic gonadotropin, chemotherapy, choriocarcinoma, fetomaternal hemorrhage, placenta

## Abstract

**Rationale:**

Near-term intraplacental choriocarcinoma (IC) coexisting with massive fetomaternal hemorrhage (FMH) is rare, and its clinical course is poorly understood. Here, we report a new case from our hospital, with detailed discussion and literature review.

**Patient concerns:**

A 21-year-old Chinese female at 35 weeks gestation was admitted to our hospital due to reduced fetal movement. Near-term IC coexisting with massive FMH was diagnosed after delivery.

**Intervention:**

The mother and infant were followed 3 months after delivery. Beta-human chorionic gonadotropin (β-HCG), pathological examination of the placenta, and computed tomography scans were performed for the mother and β-HCG was performed for the infant.

**Outcomes:**

The mother's β-HCG serum level increased from 31,280 IU/L (6 days postdelivery) to 192,070 IU/L (49 days postdelivery), and then steadily fell to 42,468 IU/L (3 months postdelivery) without chemotherapy. The mother died from metastasis and cerebral hemorrhage. The infant survived and his β-HCG serum level fell to within the normal range without chemotherapy.

**Lessons:**

FMH associated with near-term IC is a rare disease. Measurement of maternal β-HCG may therefore represent a useful parameter when IC is a possible differential diagnosis. A pathological examination of the placenta should be performed in all cases of FMH to better identify cases of IC. Future research should aim to develop methods of identifying which patients with IC should receive chemotherapy, whether we should use single- or multiagent chemotherapies, and whether there is a positive correlation between chemotherapy regimen and β-HCG serum levels.

## Abstract 2

Supplemental Digital Content is available in the text

## 1 Introduction

Intraplacental choriocarcinoma (IC), defined as choriocarcinoma in the placenta, is a rare disease with an estimated occurrence of 1 in 50,000 pregnancies^[1,2]^ and is usually diagnosed at an advanced stage following the identification of maternal metastasis. Massive fetomaternal hemorrhage (FMH), associating with IC, is an even rare occurrence. When a malignant growth of trophoblastic tissue from IC invades the uterine muscle and maternal vascular spaces, a large fetal hemorrhage may leak into the maternal circulation.^[3]^ If the fetal blood transfuses into the maternal circulation equal to or greater than 50 mL of fetal blood, we define it as massive FMH.^[4]^ Massive FMH associating with IC causes severe fetal anemia and hydrops fetalis, and represents a life-threatening complication for the fetus.^[5,6]^ Since half of patients are asymptomatic, most involved patients are uneventful and macroscopic placental review alone is of low sensitivity, it is likely that a lot of patients are missed diagnosis and the true incidence is unknown.^[2]^ Massive FMH occurs unexpectedly and the diagnosis of IC coexisting with massive FMH is often made retrospectively after fetal or intrapartum death.^[6]^ Consequently, it can represent a significant challenge for perinatology teams. To date, only 24 cases of histopathologically confirmed IC complicated FMH have been reported in the scientific literature. However, none of these existing studies have described in detail of the clinical manifestation, diagnosis, therapy, and outcomes of this uncommon complication. Furthermore, there is a lack of knowledge pertaining to the treatment of IC, particularly regarding standardized therapeutic principles. Although some cases require chemotherapy, other cases do not and simply require serum beta-human chorionic gonadotropin (β-HCG) monitoring. Consequently, prognostic factors determining which cases require chemotherapy are important to identify.

In this paper, we report a case of near-term IC coexisting with massive FMH and present a thorough review of the scientific literature of clinical manifestation, diagnostic pathways, treatment, and outcomes for both mother and baby. We also discuss the relationship between serum β-HCG levels and chemotherapy for IC.

## 2 Case presentation

**Figure 1:**
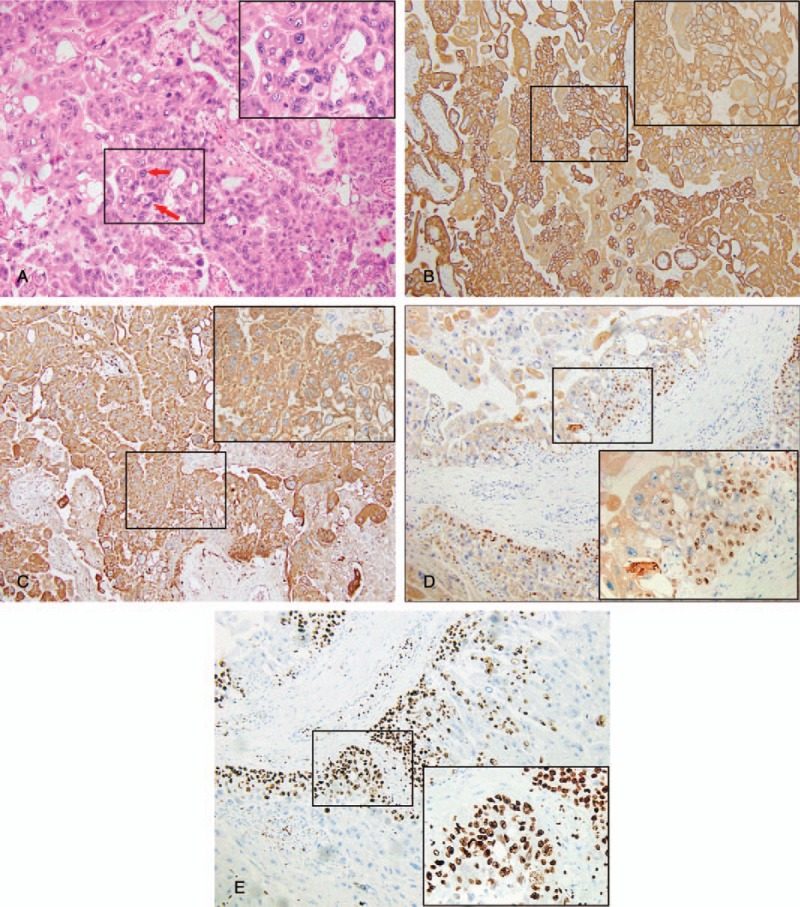
(A) Representative images of hematoxylin and eosin stain. The top right corner (400× of the image shows an enlarged image in A (100×). The arrows indicate marked cytological atypia, with variably sized nucleus. (B) Representative images of immunohistochemistry staining for CK. The top right corner (400×) of the image shows an enlarged image in B (100×). And this image shows positive expression of CK in choriocarcinoma cells. (C) Representative images of immunohistochemistry staining for HCG. The top right corner (400×) of the image shows an enlarged image in C (100×). And this image shows positive expression of HCG in choriocarcinoma cells. (D) Representative images of immunohistochemistry staining for P63. The bottom right corner (400×) of the image shows an enlarged image in D (100×). And this image shows positive expression of P63 in choriocarcinoma cells. (E) Representative images of immunohistochemistry staining for Ki-67. The bottom right corner (400×) of the image shows an enlarged image in E (100×). And this image shows positive expression of Ki-63 in choriocarcinoma cells. CK = cytokeratin, HCG = beta-human chorionic.

**Figure 2:**
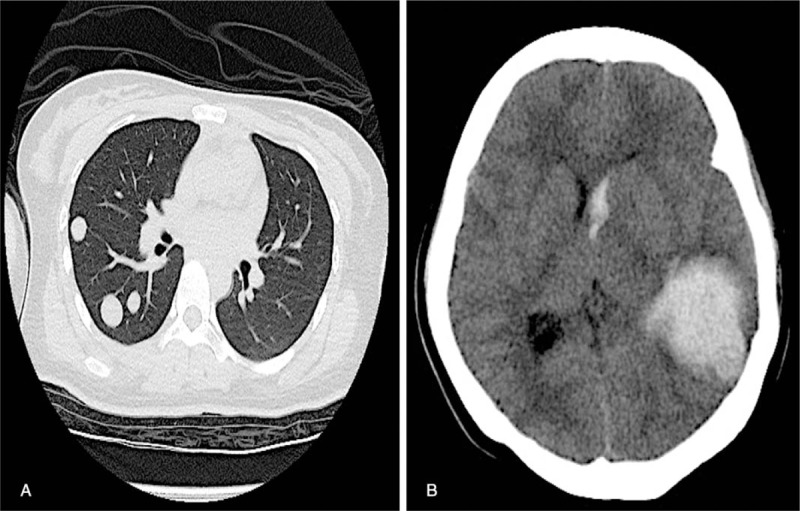
(A) Chest computed tomography (CT) scan carried out at 95 days postdelivery showing several metastatic nodules in the bilateral pulmonary tissue. (B) The cerebral CT carried out at 100 days postdelivery showing cerebral hemorrhage (left temporal lobe) and a midline shift of the brain.

A 21-year-old Chinese female (gravida 1, para 0) was hospitalized at 35 weeks of gestation due to reduced fetal movement. Abdominal examination revealed a uterine size compatible with gestational age with a fundal height of 33 cm and an abdominal circumference of 100 cm. Fetal heart rate was normal at 148 beats per minute. However, the pulse index and peak systolic flow velocity in the middle cerebral artery were 1.27 and 93.82, respectively, suggestive of fetal anemia. Fetal cardiotocography showed a sine wave. Subsequently, a cesarean section was performed and a very pale newborn male was delivered. The infant weighed 2170 g, and Apgar score was 8, 8, and 8 at the 1st, 5th, and 10th minute after delivery, respectively. Hemoglobin concentration in the umbilical artery was 2.6 g/dL; hematocrit was 10.3%. Blood types of both the mother and infant were B rhesus positive. Screening tests for congenital infection were all negative and the mother was negative for parvovirus B19 IgM. Kleihauer examination confirmed FMH. The infant was immediately given a transfusion of 82 mL type B packed red cells and responded well soon after treatment. Gross appearance of the placenta was normal. The placenta was sectioned, fixed in 10% formalin overnight, and submitted for processing. A grayish yellow nodule measuring 20 × 18 × 12 mm was found at one margin and thought to represent an infarct. Microscopic examination showed a well-circumscribed lesion composed of atypical syncytiotrophoblasts and cytotrophoblasts with geographic tumor necrosis and hemorrhage. Investigations at higher magnification showed nuclear pleomorphism and severe atypia (Fig. 1A). Immunohistochemical staining was positive for cytokeratin (Fig. 1B), HCG (Fig. 1C), protein 63 (Fig. 1D), and nuclear-associated antigen Ki-67 (15%) (Fig. 1E), but was negative for carcinoembryonic antigen, vimentin, prolactin, and cluster of differentiation 34. Combining the clinical manifestations with the results of pathological and laboratory investigations, we diagnosed the patient with an IC at 6 days postdelivery and recommended further examination and chemotherapy. However, the patient refused pelvic computed tomography (CT) scans and a chest X-ray, and refused further therapy. The patient was discharged at 7 days postdelivery. β-HCG increased from 31,280 IU/L (6 days postdelivery) to 192,070 IU/L (49 days postdelivery). She returned to our hospital at 95 days postdelivery with symptoms of coughing, and this allowed us to carry out a series of additional tests. β-HCG was 42,468 IU/L, chest CT revealed a number of metastases in the lung (Fig. 2A), and pelvic ultrasound revealed metastases in the uterus. Five days later, she was deteriorated and was admitted to the intensive care unit due to cerebral hemorrhage (Fig. 2B). Unfortunately, the patient died at 109 days postdelivery. The International Federation of Gynecology and Obstetrics^[7]^ score was 4 at 6 days postdelivery. However, it was changed to 9 at 95 days postdelivery.

From a neonatal perspective, 6 days after birth, the infant's β-HCG level was 30.4 IU/L (normal range <0.2 IU/L). He was discharged on day 10 without any specific chemotherapy. We monitored the baby after discharge and his β-HCG level returned to normal by day 41. At the time of writing, the baby remains free from disease. This case presentation was consented by the patient's husband.

## 3 Discussion

Benson et al^[3]^ described the first case of massive FMH in 1962 as a complication of IC. Thereafter, several reports have documented an association between IC and FMH. The incidence of IC was reported to be approximately 1 in 50,000 pregnancies. However, the true incidence of IC remains largely unknown because microscopic placental examination is not routinely performed in all complicated pregnancies with FMH or fetal distress. The pathogenesis of IC and FMH remains to be determined, although several hypotheses have been put forward to explain the coexistence of choriocarcinoma and normal pregnancy.^[8]^

We reviewed the existing literature describing 24 previous cases (Table S1) and related these findings to the current case. The mean age of these patients was 28.87 ± 6.62 years, and most patients delivered their babies at term. Eleven pregnancies resulted in delivery by cesarean section due to fetal distress, and another was induced for caesarian delivery due to hypertension. Eleven of these earlier patients were asymptomatic when diagnosed. Ten patients had metastasis; 1 patient died due to cerebral involvement despite the fact that she had received chemotherapy. Our case did not have metastasis when diagnosed at 6 days postdelivery. However, the patient refused to undergo further imaging or therapy. When she returned to our hospital 3 months later, she had developed metastasis in several organs (uterus, lung, and cerebral) and subsequently died due to cerebral hemorrhage.

Of the patients listed in Table S1, there were 6 intrauterine fetal death cases. Fifteen (83.3%) of the 18 live births had hemoglobin level less than 9 g/dL. Of the 5 cases with fetal or neonatal metastasis, all died in uterine or during newborn period. In this current case, the newborn had a good prognosis despite increased β-HCG levels on day 6 after birth; β-HCG subsequently returned to normal levels without chemotherapy. In our study (including our own case), we found that the metastasis of the fetus was 20% (5/25), while in the study by Jiao et al^[2]^ was only 5.3% (2/38). We speculate that this might be due to the fact that we included IC complicated FMH patients only while Jiao et al included all IC cases, whether coexisted with FMH or not.

In addition, within the 25 cases, except 4 cases which did not describe fetal gender, there were 6 female fetuses and 15 male fetuses. The male fetuses were more than 2 times as many as the female fetuses. However, according to what we have searched, there was not any study about the association between fetal gender and IC coexisting with FMH. Similarly, whether IC patient with male fetus was prone to complicate FMH, there was not any research on this. Hence, further research could aim to verity if IC patients with male fetus are more likely to cause FMH.

In over half of the cases reviewed, the placenta was not sent for pathological analysis, largely owing to the rarity of this condition and the fact that clinicians were unsuspecting of such problems.^[9]^ Consequently, a golden opportunity for early diagnosis was missed. Most cases presented in the weeks or months following parturition, while the time between delivery and diagnosis ranged from day 1 to 3 months. Subsequently, it would be difficult to detect IC in the uterus. Hysterectomy and chemotherapy are the usual choice for therapy. Chemotherapy is particularly popular as it significantly reduces mortality. Of the 24 cases reviewed here, 15 received serum β-HCG monitoring and had detailed data recorded. Eight of the 15 patients were without metastasis (Table S1). The serum β-HCG levels among these 8 patients were 11.311 to 766, 433 IU/L. Five of these 8 patients received chemotherapy, while the other 3 just received serum β-HCG monitoring. All of these 8 patients had good prognosis. As the 7 patients with metastasis, the serum β-HCG levels were 986 to 2,000,000 IU/L, 4 of the 7 were over 500,000 IU/L. Five of the 7 patients received multiagent chemotherapy. Among the 7 patients with metastasis, 1 patient died 60 days postpartum. The serum β-HCG of this patient was 2 million IU/L at diagnosis. Among the 9 patients without detailed serum β-HCG results, 6 achieved spontaneous remissions without chemotherapy, and 1 had a good prognosis following total hysterectomy without chemotherapy. Among these 24 patients, 9 of 24 just received serum β-HCG monitoring and had good prognosis. Follow-up examination of our case revealed that serum levels of β-HCG increased from 31,280 IU/L (6 days postdelivery) to 192,070 IU/L (49 days postdelivery), but then fell automatically to 42,468 IU/L (3 months postdelivery) without any therapeutic intervention. However, the mechanisms responsible for this phenomenon remain unclear.

β-HCG is well known as a useful marker for trophoblastic-derived tumors such as choriocarcinoma. Some reports recommended that if there was no elevation in the level of serum β-HCG then chemotherapy was not necessary when the mother was diagnosed with IC.^[10]^ However, some patients died without chemotherapy, as was the case in our patient. Therefore, in cases of IC, methods of identifying which patients require chemotherapy must be determined. In addition, future studies should aim to establish whether single- or multiagent chemotherapy is required, or whether there is a positive correlation between chemotherapy regimen and serum β-HCG levels. Our present case demonstrated that FMH should always be followed by a detailed examination of the placenta and serial serum-HCG monitoring of both mother and child. This practice should help to diagnose the early signs of possible malignant dissemination.

In conclusion, IC coexisting with FMH is a rare disease. Measurement of maternal β-HCG might represent a useful parameter for the chemotherapy of IC. A pathological examination of the placenta should be performed in all cases of FMH to better diagnose cases of IC. Future research should aim to develop methods of identifying which patients with IC complicated FMH should receive chemotherapy, whether we should use single- or multiagent chemotherapies, and whether there is a positive correlation between chemotherapy regimen and serum β-HCG levels.

## Author contributions

All the authors participated in the formulation of the methodology for this research.

**Conceived and designed the experiments:** S.W. Wen, Q. She, Z. Cheng, X.Y. Guo.

**Contributed reagents/materials/analysis tools:** Q. She.

**Performed the experiments:** Q. She, Z. Cheng, F. Luo.

**Analyzed the data and wrote the paper:** S.W. Wen, Q. She, D. El-Chaar.

**Conceptualization:** Q. She, S.W. Wen, X. Guo, Z. Cheng.

**Data curation:** Q. She, Z. Cheng.

**Formal analysis:** Q. She, S.W. Wen, X. Guo, Z. Cheng.

**Funding acquisition:** S.W. Wen.

**Investigation:** Q. She, X. Guo, Z. Cheng.

**Methodology:** D. El-Chaar, F. Luo, Q. She, S.W. Wen, X. Guo, Z. Cheng.

**Project administration:** F. Luo, Q. She.

**Software:** Q. She.

**Validation:** D. El-Chaar.

**Visualization:** D. El-Chaar.

**Writing – original draft:** Q. She, S.W. Wen.

**Writing – review & editing:** D. El-Chaar, S.W. Wen.

## Acknowledgements

The authors thank the patient's family member for allowing present this case, and the staff of the Obstetrics Department of the Six Affiliated Hospital, Guangzhou Medical University, China for their assistance with data collection.

## Supplementary Material

Supplemental Digital Content
